# Ketogenic Diet: A Role in Immunity?

**DOI:** 10.15844/pedneurbriefs-34-5

**Published:** 2020-03-03

**Authors:** Andrea C. Pardo

**Affiliations:** 1 Division of Neurology, Ann & Robert H. Lurie Children’s Hospital of Chicago, IL; 2 Departments of Pediatrics and Neurology, Northwestern University Feinberg School of Medicine, Chicago, IL

**Keywords:** Ketogenic Diet, Immunity, Epilepsy

## Abstract

Investigators from The Department of Comparative Medicine, from Yale School of Medicine report the effect of the ketogenic diet on the T cell immune function in mice exposed to influenza virus.

Investigators from The Department of Comparative Medicine, from Yale School of Medicine report the effect of the ketogenic diet on the T cell immune function in mice exposed to influenza virus. The investigators examined transgenic immunocompetent mice exposed to an intranasal challenge of influenza virus. The mice were fed ketogenic diet prior to the infection. The team studied survival and weight loss on the mice. This study noted that mice that were fed ketogenic diet had better survival and decreased loss when compared to mice that were not exposed to the ketogenic diet. The investigators sought to analyze the molecular underpinnings of this finding. They performed transcriptome analysis of the mice’s lungs noting that several genes were upregulated in ketogenic diet fed mice; particularly genes associated to δ T cell responses were increased; including number of cells in lung tissue, and release of cytokines related to this specific cell type. The mice consistently showed lower virus titers than controls. The investigators then tested whether the ketogenic diet was actually exerting an effect on these T cell populations by comparing the function in mice fed a non-ketogenic high caloric density diet. They noted that these effects were specific for the ketogenic diet fed animals only. Additionally, the investigators noted that the ketogenic diet increased δ T cell proliferation, noting that this was not directly related to increases in β hydroxybutyrate, the primary energy substrate during glucose deprivation associated to ketogenic diet, but rather finding that the effect on δ T-cell population is mediated by favoring fatty acid oxidation. This paper concludes that the effect of ketogenic diet exerts a potentially previously unrecognized immune effect. [1]

COMMENTARY. The ketogenic diet has been used since the early twentieth century for the treatment of epilepsy. There are multiple mechanisms by which the ketogenic diet exerts its anticonvulsive effect. The increased levels of ketone bodies such as β-hydroxybutyrate have shown to be protective of injury by oxygen reactive species. The modulation and metabolism of GABA systems may also be implicated in the antiseizure mechanism of the ketogenic diet. Additionally, enhanced excitatory neurotransmitter metabolism, effects on synaptic transmission and energy metabolism may be associated to its antiepileptic effect [2]. The role of the ketogenic diet in the treatment of super-refractory status epilepticus has been reported in recent literature, particularly its efficacy in immune mediated encephalitis as well as febrile infection-related epilepsy syndrome (FIRES), and new onset refractory status epilepticus (NORSE) [3]. The efficacy of the ketogenic diet on the treatment of this immune mediated status epilepticus may be mediated by the systemic and metabolic effects of the ketogenic diet on the immune system [4]. Further studies are required to determine the direct mechanisms by which the ketogenic diet affects inflammation and immunity. The current study highlights molecular pathways of modulation of the immune system that may potentially be harnessed for the treatment of epilepsy. Additionally, it highlights the systemic effects of ketogenic diet identifying a novel immune modulating mechanism

## Disclosures

The author has declared that no competing interests exist.
